# SRC family kinase FYN promotes the neuroendocrine phenotype and visceral metastasis in advanced prostate cancer

**DOI:** 10.18632/oncotarget.6398

**Published:** 2015-11-26

**Authors:** Murali Gururajan, Karen A. Cavassani, Margarit Sievert, Peng Duan, Jake Lichterman, Jen-Ming Huang, Bethany Smith, Sungyong You, Srinivas Nandana, Gina Chia-Yi Chu, Sheldon Mink, Sajni Josson, Chunyan Liu, Matteo Morello, Lawrence W. M. Jones, Jayoung Kim, Michael R. Freeman, Neil Bhowmick, Haiyen E. Zhau, Leland W.K. Chung, Edwin M. Posadas

**Affiliations:** ^1^ Urologic Oncology Program/Uro-Oncology Research Laboratories, Samuel Oschin Comprehensive Cancer Institute, Cedars-Sinai Medical Center, Los Angeles, CA 90048, USA; ^2^ Division of Hematology/Oncology, Department of Medicine, Cedars-Sinai Medical Center, Los Angeles, CA 90048, USA; ^3^ Cancer Biology Program, Departments of Surgery, Medicine, and Biological Sciences, Cedars-Sinai Medical Center, Los Angeles, CA 90048, USA; ^4^ Urological Research, Huntington Medical Research Institutes, Pasadena, CA 91101, USA

**Keywords:** SRC kinase, NEPC, metastasis, neuroendocrine, prostate cancer

## Abstract

FYN is a SRC family kinase (SFK) that has been shown to be up-regulated in human prostate cancer (PCa) tissues and cell lines. In this study, we observed that FYN is strongly up-regulated in human neuroendocrine PCa (NEPC) tissues and xenografts, as well as cells derived from a NEPC transgenic mouse model. *In silico* analysis of FYN expression in prostate cancer cell line databases revealed an association with the expression of neuroendocrine (NE) markers such as CHGA, CD44, CD56, and SYP. The loss of FYN abrogated the invasion of PC3 and ARCaP_M_ cells in response to MET receptor ligand HGF. FYN also contributed to the metastatic potential of NEPC cells in two mouse models of visceral metastasis with two different cell lines (PC3 and TRAMPC2-RANKL). The activation of MET appeared to regulate neuroendocrine (NE) features as evidenced by increased expression of NE markers in PC3 cells with HGF. Importantly, the overexpression of FYN protein in DU145 cells was directly correlated with the increase of CHGA. Thus, our data demonstrated that the neuroendocrine differentiation that occurs in PCa cells is, at least in part, regulated by FYN kinase. Understanding the role of FYN in the regulation of NE markers will provide further support for ongoing clinical trials of SFK and MET inhibitors in castration-resistant PCa patients.

## INTRODUCTION

Over 90% of prostate cancers (PCa) occur in the form of adenocarcinomas, which are characterized by dysregulated growth of the epithelial cells that typically secrete prostate specific antigen (PSA). Many of these are tractable when treated with currently available therapies even though nearly every PCa contains a subpopulation of neuroendocrine (NE) cancer cells scattered throughout the tumor that make up 1% or less of the total tumor volume [1, 2].

In some cases of PCa, patients exhibit a clinical phenotype dominated by NE behavior. These NE prostate cancers (NEPCs) do not typically express androgen receptor (AR). Because PSA is a target gene of AR, patients with NEPC typically have very low serum PSA concentrations. Clinically, NEPCs exhibit aggressive metastatic properties leading to disease spread to visceral organs such as the liver and lung. This pattern of clinical behavior has been strongly associated with shortened overall survival [3, 4]. NEPC is distinguished by the expression of markers including chromogranin A (CHGA), chromogranin B (CHGB), synaptophysin (SYP), CD44, and CD56 [5]. In addition, AURKA and MYCN amplifications in primary prostatic adenocarcinoma have been described to predict the differentiation of NEPC [6, 7]. Since the introduction of next-generation AR-inhibitors, there appears to have been an increase in the incidence of NEPC, which is thought to arise during the development of resistance. NEPCs are typically treated with cytotoxic chemotherapy with platinum-containing regimens, but these therapies are non-curative and relatively toxic. As such, they represent an urgent and unmet clinical and translational problem.

We have determined that the FYN kinase (one of the nine identified SFKs) is overexpressed in PCa [8–10]. Our published studies have shown that FYN plays an important role in cellular motility in cancer [9], particularly when driven by hepatocyte growth factor (HGF), which is found in abundance in the plasma of patients with both acinar prostatic adenocarcinomas and NEPC [9, 11, 12]. Data from our group and others have demonstrated particular importance of FYN and other SFKs in later events in PCa progression. However, these studies did not directly address the role of FYN in NEPC. The role of SFKs, particularly the FYN kinase, in NEPC has not been characterized. *Fyn* knockout mice develop neurological defects such as blunted long-term potentiation (LTP), impaired special learning, and altered hippocampal development, suggesting a neuronal role for FYN kinase and a potential role in cancers that have NE features [13]. Recent evidence suggests that nerves innervate the prostate microenvironment in unique fashion. Moreover, there is evidence to show that neuronal cells and endocrine factors promote tumor generation and progression of NEPC [14].

In the present study, FYN kinase expression was associated with neuroendocrine biomarkers in PCa cell lines and PCa liver metastasis derived cells. *In vitro* and *in vivo* data demonstrate that FYN promoted the invasion and metastasis of NEPC cells. Together, these data highlight the importance of FYN in the regulation of NE markers, NEPC invasion and metastasis.

## RESULTS

### FYN is overexpressed in NEPC cell lines and tissues

**Figure 1:**
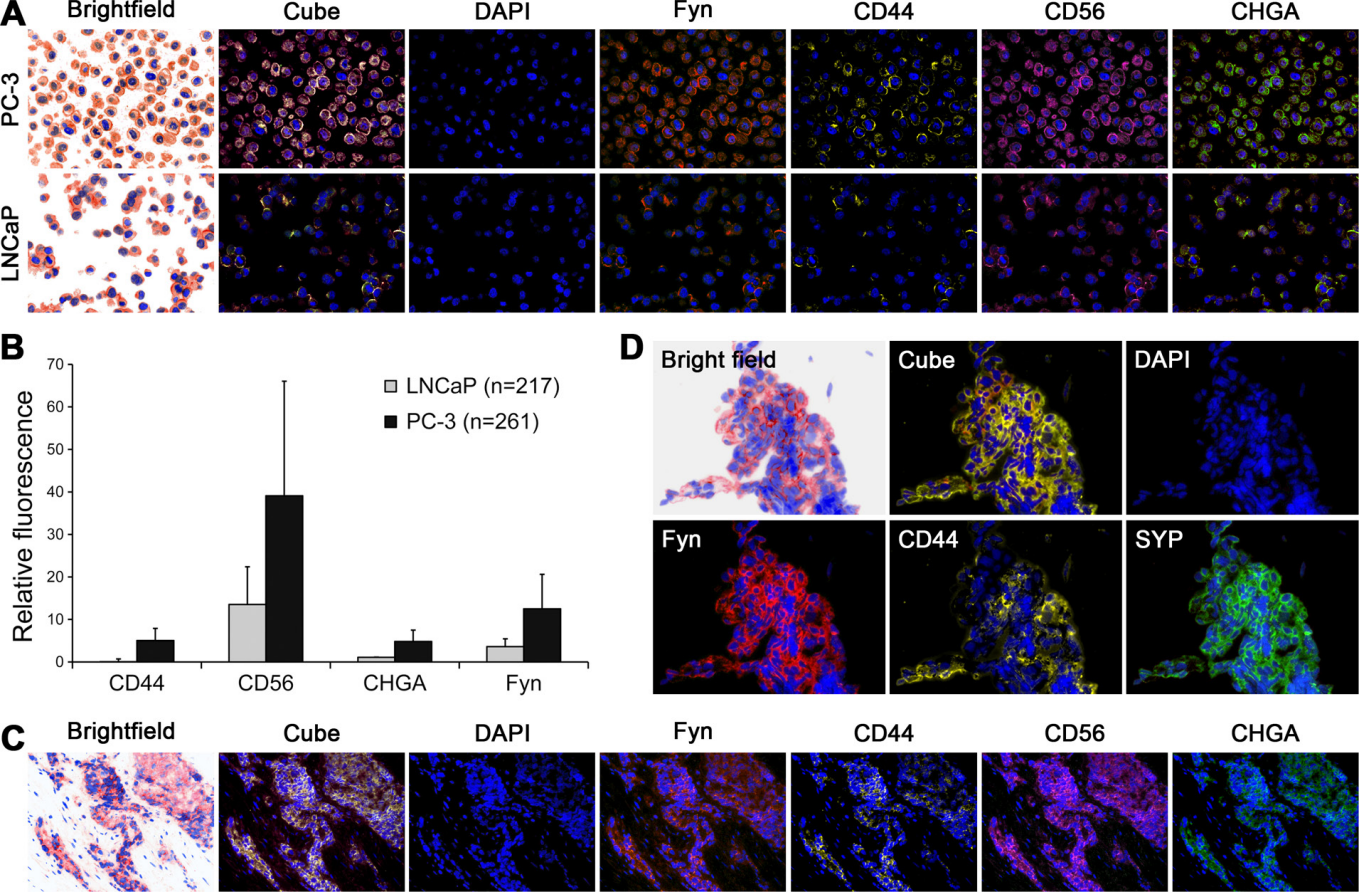
FYN kinase co-expressed with neuroendocrine biomarkers in primary PCa with neuroendocrine phenotype and in PCa liver metastasis Analysis of NE markers (CD44, CD56, and CHGA) and FYN in cell and tissues were performed by multiplexed quantum dot labeling method. (**A**) Metastatic human PC3 cells expressed higher FYN, and NE markers than the indolent LNCaP cells. (**B**) Relative fluorescence quantification of FYN and NE biomarkers in PC-3 and LNCaP cells. (**C** and **D**) Biopsy samples from patients with neuroendocrine PCa: (C) Primary tumor (D) Pleural biopsy.

Our previous studies identified that FYN expression is increased in PCa [9] although FYN kinase is typically associated exclusively with neuronal activity. This observation led us to hypothesize that FYN expression might be detectable in a subset of PCa with NE features. Accordingly, Huang and colleagues have reported that the PC3 cell line is a bonafide prostatic small cell carcinoma with NE features [15]. In the present study, we examined PC3 cells for FYN expression and observed that PC3 cells have greater expression of FYN compared to LNCaP cells (a more acinar or non-NE cell line) consistent with our previous published observations [9] (Figure 1A and 1B). FYN expression correlated with the expression of markers of NE differentiation (Figure 1A and 1B) and QD analysis of human PCa patient tissues expressing NE markers including CHGA, CD44, CD56, and SYP confirmed co-expression of FYN (Figure 1C and 1D). In particular, FYN expression was approximately 4-fold higher in NEPC patient tissues compared with a standard adenocarcinoma. Together, these observations suggested that there was a strong correlation between FYN and NEPC.

### FYN expression is associated with NE marker expression in PCa

**Figure 2:**
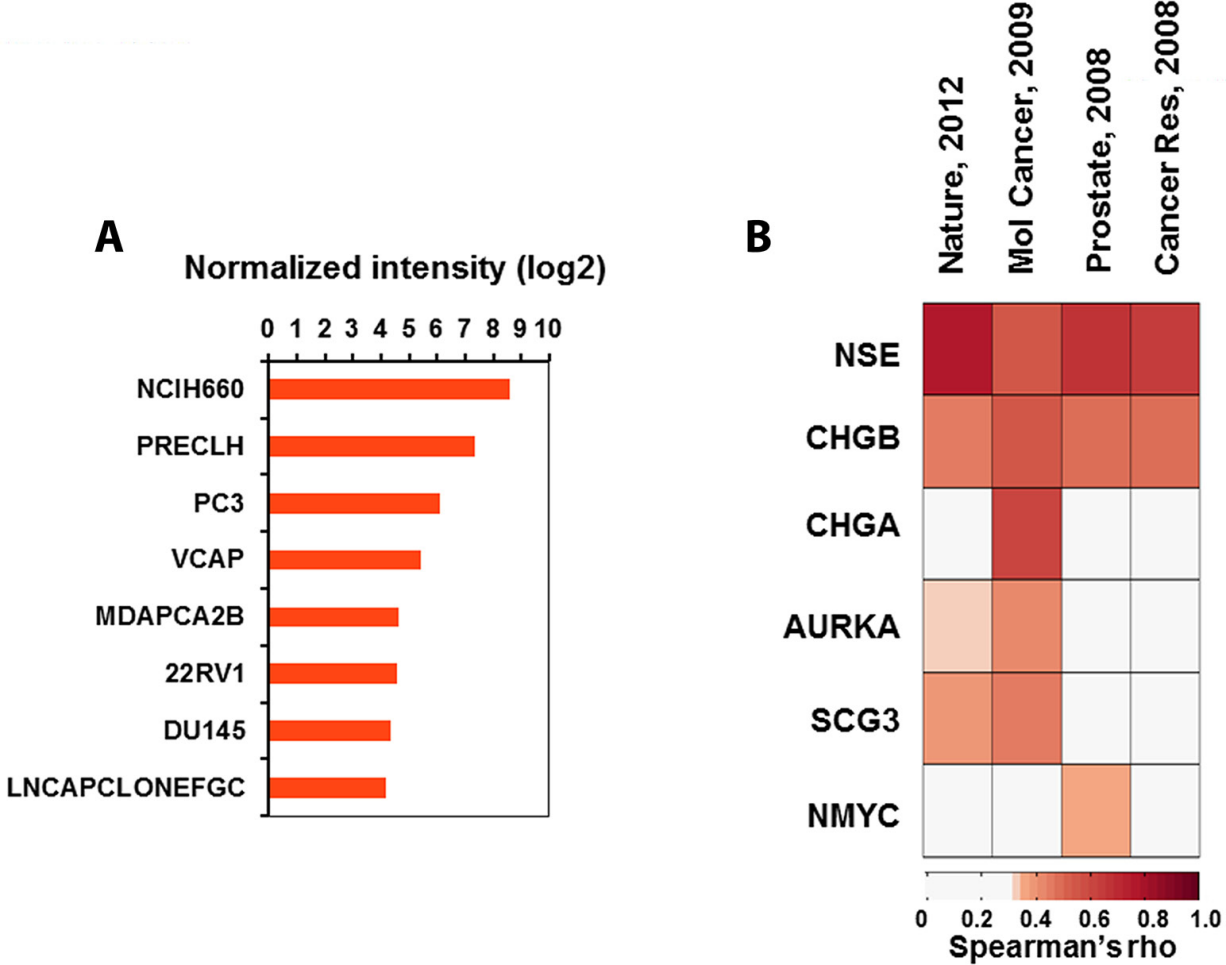
Association analysis of FYN expression with NE phenotype (**A**) Bar graphs showing normalized intensities of FYN in 8 PCa cell lines characterized in CCLE. (**B**) Heatmap showing Spearman's rho of FYN with expression of NE biomarkers in four independent datasets derived from human PCa tissues.

We next examined whether FYN expression was associated with NE tumor marker expression lines cataloged in the Cancer Cell Line Encyclopedia (CCLE, http://www.broadinstitute.org/ccle). Analysis of mRNA expression across the CCLE lines revealed that FYN was expressed at higher levels in the cell lines derived from the tumors such as neuroblastoma, small cell lung cancer, and medulloblastoma. Although the PCa cell lines included in the CCLE were characterized with low expression of FYN, when compared to most of the NE cell lines, this was not unexpected as the majority of cell lines used in PCa research are of an acinar adenocarcinoma phenotype. However, NCI-H660 cells (a well-defined NEPC cell line [16, 17]) showed the highest expression of FYN and PC3 showed third highest expression among the 8 PCa cell lines in CCLE (Figure 2A). The correlation between FYN and NE markers including NSE, CHGA, CHGB, AURKA, SCG3, and MYCN was next analyzed using gene expression profiles obtained from four public datasets [18–21]. All NE markers showed significant correlation with FYN in at least one of the datasets (Figure 2B).

### FYN regulates growth and invasion of PC3 and ARCaP_M_ cells *in vitro*

**Figure 3:**
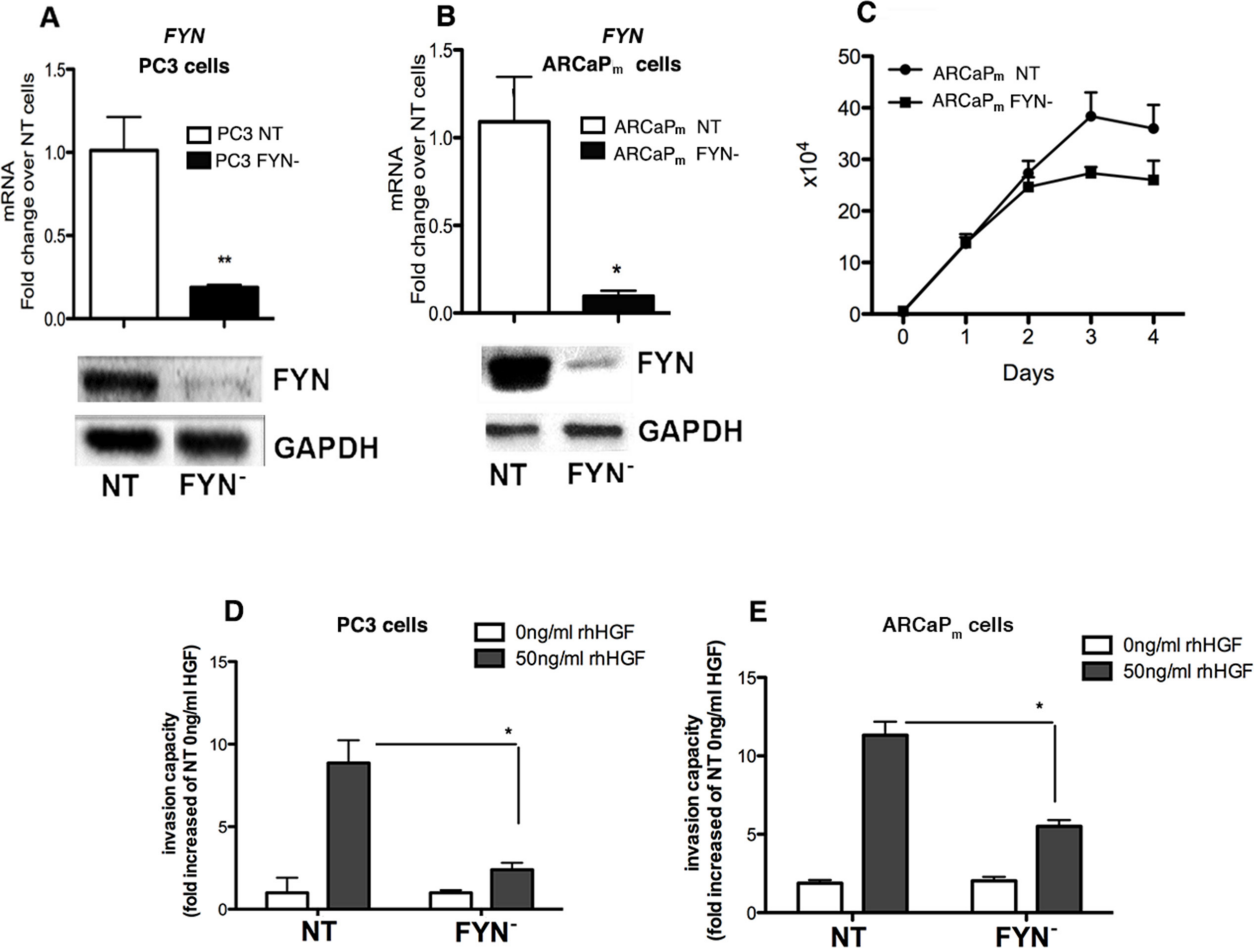
FYN promotes invasion of PCa cells *in vitro* in response to HGF stimulation (**A–B**) RT-PCR assays and Immunoblot of FYN expression in PC3 and ARCaP_M_ variants confirming decreased expression of FYN in the knockdown line. (**C**) 4-day growth curves comparing ARCaP_M_ FYN- to ARCaP_M_ NT. Error bars represent standard error of the mean with 3 replicates for each day. (**D–E**) Matrigel invasion assays of PC3 (NT and FYN-) at 16 hours or ARCaP_M_ (NT and FYN-) at 48 h post incubation with or without 50 ng/ml of rhHGF. Following incubation, the cells that had invaded and attached to the lower surface of the membrane were fixed and stained with hematoxilin. Cell numbers were counted in 4 different randomly chosen microscope field per membrane and analyzed using Image J software. Duplicates were performed. Data are representative of two independent experiments. *p < 0.05, **p < 0.01 when FYN- was compared with NT.

The impact of FYN on the growth and invasive potential of PCa cells was determined using standard Matrigel invasion assays. To perform these assays, we generated PCa cell lines in which FYN was depleted via shRNA targeting approaches. FYN knockdown was confirmed by RT-PCR and Western blot analysis (Figure 3A and 3B). The relative mRNA expression of *FYN* shows that the total *FYN* levels in PC3 are significant higher when compared with ARCaP_M_ cells. Also, *FYN* levels on PC3 and ARCaP_M_ are significantly higher than DU145 cells (Supplementary Figure 1).

We next analyzed the role of FYN in the proliferation index of ARCaP_M_. The lack of FYN impairs the full ability of the cells to proliferate when compared with NT cells (intact FYN control). After 4 days of culture, there was a 28% decrease on ARCaP_M_ FYN- cells growth when compared with ARCaP_M_ NT cells (Figure 3C). In addition, we observed a decrease in invasive capacity in response to HGF-stimulation for the FYN depleted cells as compared to their corresponding controls (Figure 3D and 3E). These findings demonstrate that FYN activation regulates PCa cell invasion not only in PC3 cells but in ARCaP_M_ cells as well.

### FYN promotes visceral metastasis of NEPC cells *in vivo*

**Figure 4:**
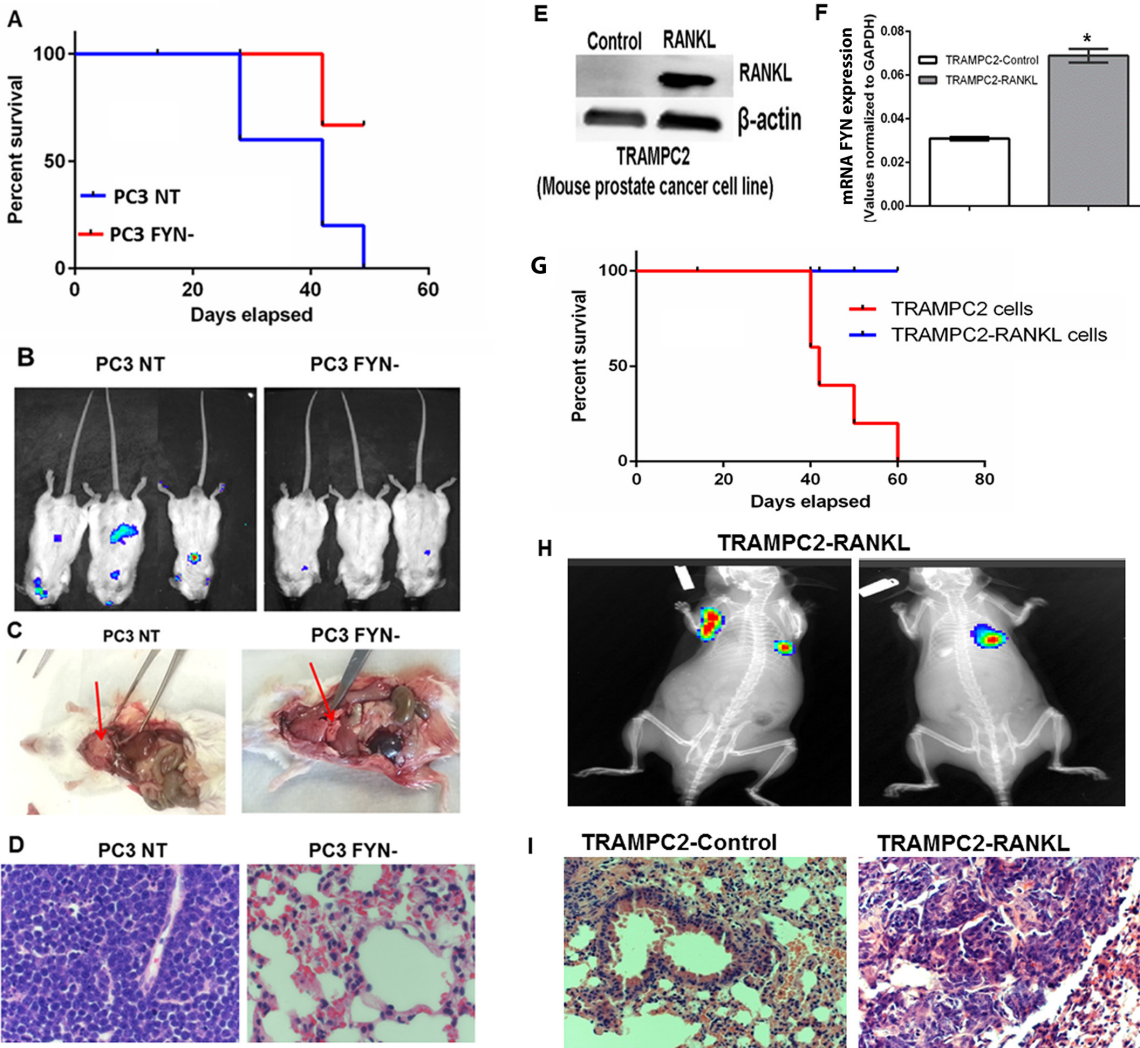
FYN promotes invasion and metastasis of NEPC cells *in vivo* Luciferase tagged PC3 cells were introduced into SCID mice via intracardiac injection. (**A**) Kaplan-Meier survival curve of mice (*n* = 5/group) injected with PC3 NT and PC3 FYN-. (**B**) Bioluminescent signal of PC3 NT and PC3 FYN- at 4 weeks after intracardiac injection. (**C**) Necropsy analysis and (**D**) Histological analysis of lungs from PC3 NT and PC3 FYN- intracardiac injections in SCID mice. (**E**) Western blotting of RANKL expression, and (**F**) RT-PCR analysis of FYN expression of TRAMPC2 -Control and TRAMPC2-RANKL cells *p < 0.05. (**G**) Kaplan-Meier survival curve of C57BL/6 mice (*n* = 5/group) injected with TRAMPC2 and TRAMPC2-RANKL cells. (**H**) Bioluminescence signal and (**I**) H&E sections of lungs from mice injected with TRAMPC2 and TRAMPC2-RANKL cells at day 30 post-injection.

On the basis of the above *in vitro* work and our previous studies [9], we hypothesized that FYN depletion would reduce the metastatic potential in tumor cells. To address this hypothesis, luciferase-tagged PC3 NT and PC3 FYN- were introduced into SCID mice via intracardiac injection and tracked periodically by bioluminescent imaging. Mice injected with PC3 FYN- cells developed fewer metastatic lesions and exhibited increased survival when compared to mice injected with PC3 NT cells (Figure 4A and 4B). Mice that were inoculated with PC3 NT cells became extremely moribund and had to be sacrificed at earlier time points as shown in the Kaplan-Meier Survival Curve Analysis (Figure 4A). At 30 days after inoculation, we observed an increase in tumor growth in the control group but not in the PC3 FYN- group (Figure 4B). Interestingly, non-osseous metastases were detected by imaging, indicating that FYN expression promoted visceral metastasis. This observation was confirmed at necropsy during which tumors were detected within the lung parenchyma (Figure 4C). Histopathological analysis revealed massive tumor cell infiltration in the lungs of mice inoculated with PC3 NT cells with near loss of lung architecture (Figure 4D). Conversely, the lung architecture was intact in mice that received PC3 FYN- cells, suggesting that FYN expression in NEPC cells was responsible for metastatic colonization (Figure 4D).

In a parallel study, we stably expressed RANKL, a bone tropic factor in TRAMPC2 cells [22–26] derived from the TRAMP model of murine PCa (TRAMPC2-RANKL). Transcript levels showed that TRAMPC2-RANKL cells overexpress *FYN* (Figure 4F), *SYP* and *CHGA* (Supplementary Figure 2). The inoculation of this tumor line via the intracardiac route led to metastasis and lethality in immune intact C57BL/6 mice (Figure 4G–4H). Histological analysis showed that the tumor load was much greater in the lungs of mice that received TRAMPC2-RANKL compared to the control group. (Figure 4I). Thus, these results demonstrate that FYN is highly expressed in NEPC cells and appears to dictate the propensity to PCa to metastasize to visceral organs including the lung.

### FYN expression is directly associated with the regulation of NE markers

**Figure 5:**
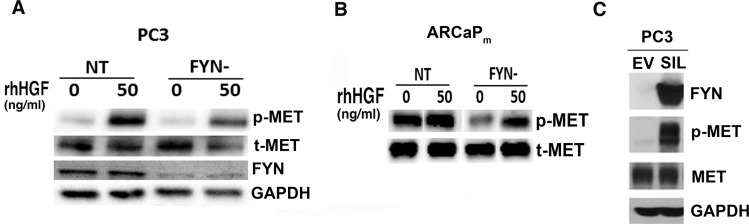
FYN promotes MET activation and phosphorylation in PC3 and ARCaP_M_ cells (**A–B**) Immunoblot analysis of the phospho-MET profile of NT and FYN- cells after 20 minutes of rhHGF stimulation. (**C**) phospho-MET profile of PC3 FYN- EV, PC3 FYN- SIL cells without rhHGF stimulation.

Recognizing that HGF is a growth factor found in excess in the plasma of patients with NE cancers, and the *in vitro* phenotypes of HGF-stimulated PCa cells (see Figure 3D and 3E) [9], we next examined the impact of FYN on MET activation. To characterize the relationship between FYN and MET using FYN-manipulated lines, FYN knockdown was used to suppress MET activation. In fact, PC3 and ARCaP_M_ FYN- cells have an attenuated ability to phosphorylate MET (Figure 5A and 5B). Next, using a PC3 subline with rescued expression of FYN (PC3 FYN- SIL) and a control line containing GFP (PC3 FYN- EV), it was apparent that the overexpression of FYN in PC3 cells led to the restoration of MET phosphorylation (Figure 5C).

**Figure 6:**
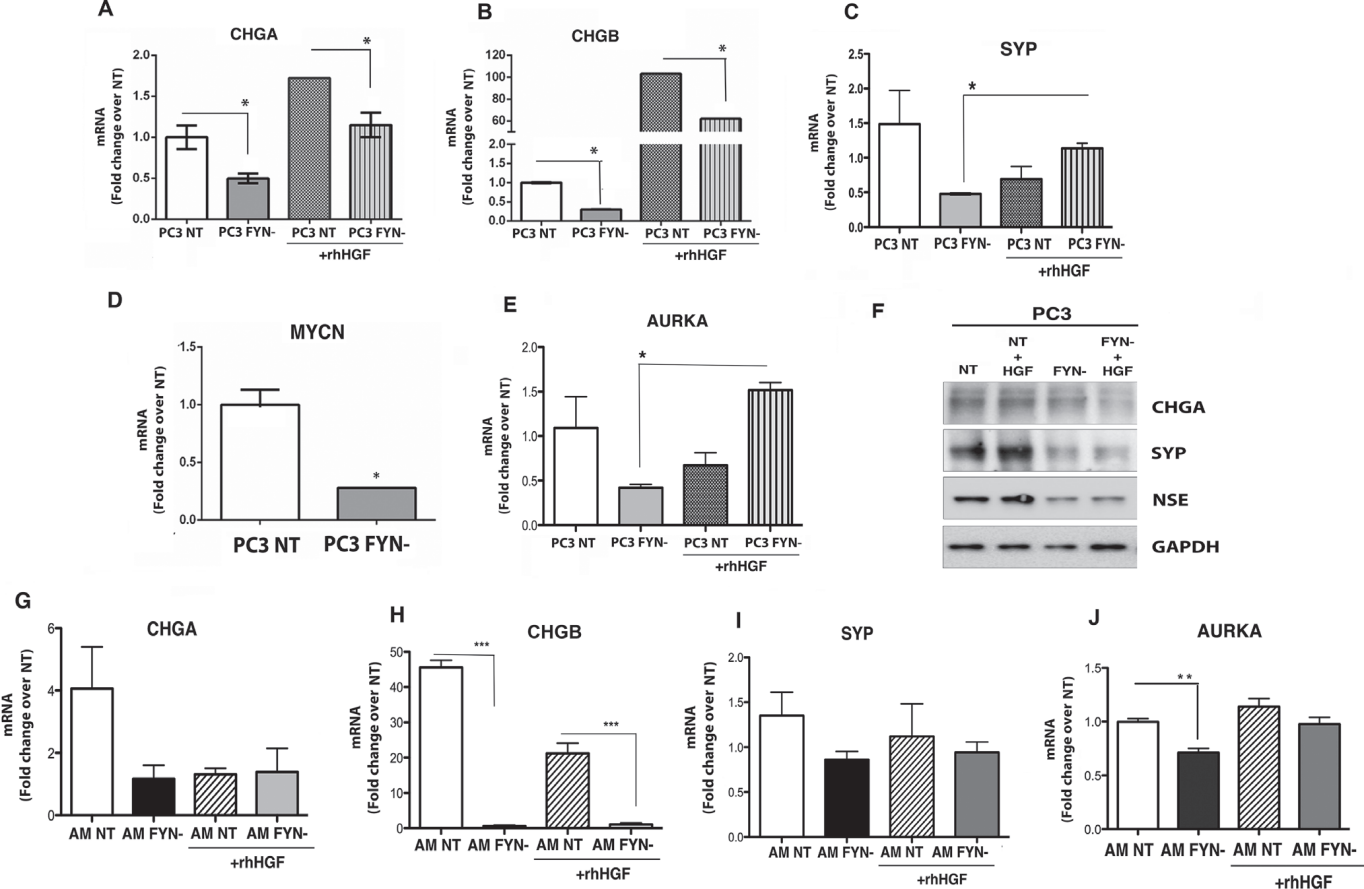
FYN signaling regulates the expression of NE markers in PC3 and ARCaP_M_ cells PC3 and ARCaP_M_ cells were starved overnight on 0.1% BSA RPMI or T medium, respectively. Cells were stimulated with or without 50 ng/ml of rhHGF for 20 min. (**A–E**) Quantitative real-time PCR (TaqMan) for NE markers (*CHGA, CHGB, SYP, and AURKA*) in PC3 NT and FYN- cells (+/− HGF stimulation). (**F**) Protein expression of CHGA, SYP, and NSE in PC3 NT (+/− HGF), PC3 FYN- (+/− HGF). (**G–J**) Transcript levels of *CHGA, CHGB, SYP* and *AURKA* on ARCaP_M_ cells. Data represent the means ± SEM. Statistically significant differences are indicated (*p < 0.05), **p < 0.01; ***p < 0.0001).

We then asked if there was a correlation between FYN/MET signaling axis and the regulation of NE markers in PCa cells. To answer this question, we analyzed the expression of NE markers, CHGA, CHGB, SCG3, SYP, MYCN, AURKA, and NSE in PC3 NT and PC3 FYN- cells. In the absence of HGF, PC3 FYN- cells exhibited lower expression of NE markers (Figure 6A–6F). The addition of HGF increased MET phosphorylation and resulted in a significant increase in mRNA levels of *CHGA, CHGB* on PC3 cells (Figure 6A–6E). Protein levels of CHGA, SYP and NSE were analyzed by Western blot (Figure 6F). Furthermore, HGF stimulation increased *SYP* and *AURKA* in PC3 FYN- suggesting that MET activation may have an indirect effect on NE markers and it is independent of FYN expression in the PC3 system (Figure 6).

The analysis of NE markers was performed in ARCaP_M_ cells (Figure 6G–6J). As observed in PC3 cells, the knockdown of FYN expression inhibited the transcript levels of *CHGA, CHGB, SYP*, and *AURKA*. Specifically on these cells, the regulation of NE markers was dependent of FYN but independent of MET phosphorylation.

**Figure 7:**
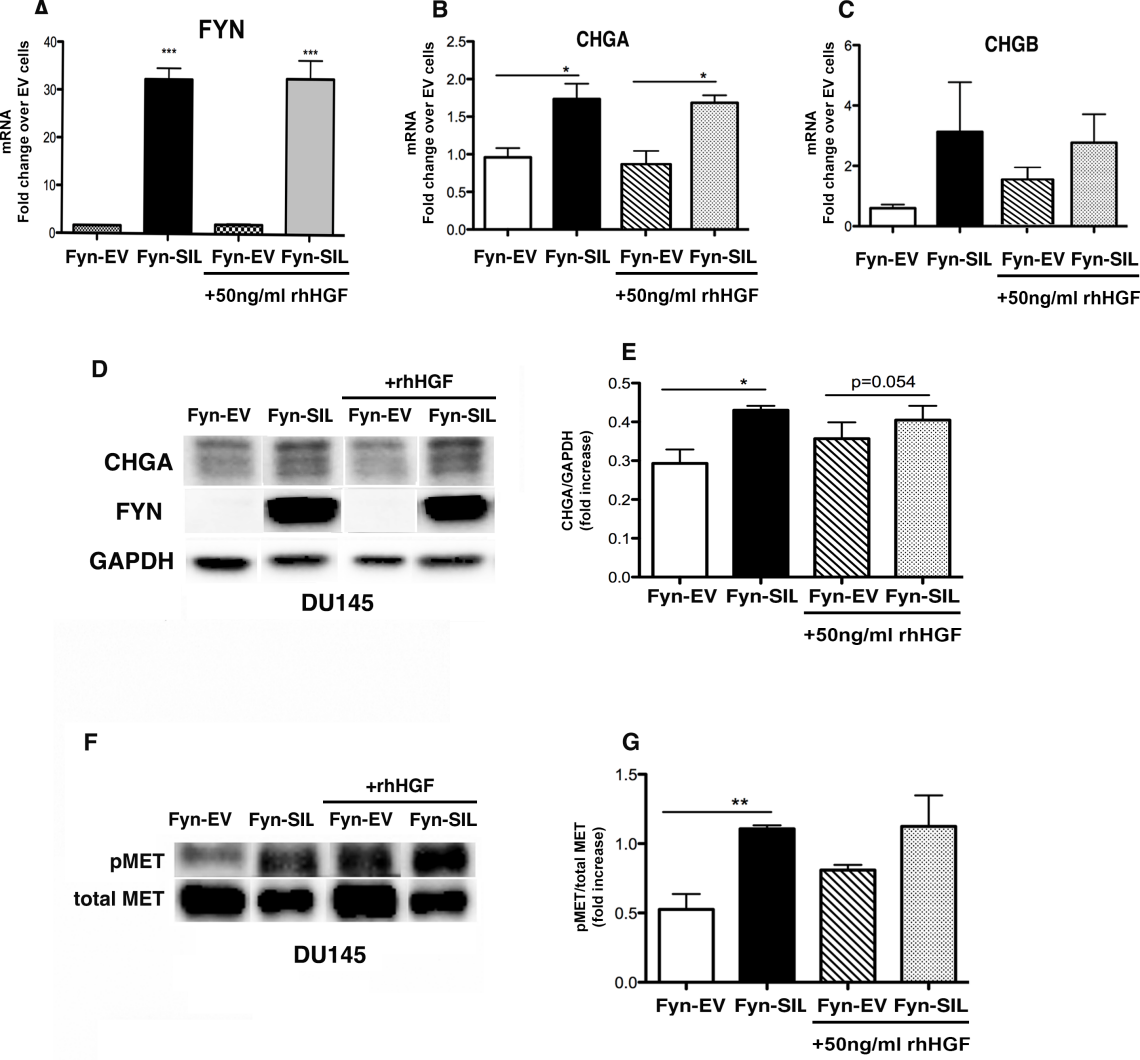
FYN expression is directly associated with NEPC phenotype DU145 Fyn-EV and DU145 Fyn-SIL were starved overnight on RPMI 0.1% BSA. 50 ng/ml of rhHGF was added for 20 min and cells were analyzed. (**A–C**) Transcript levels of *FYN, CHGA*, and *CHGB* by Taqman assay. Data represent the mean ± SEM of triplicate wells. (**D–G**) Immunoblots of DU145 Fyn-EV and DU145 Fyn-SIL (+/− rhHGF) show the expression of CHGA, FYN, pMET and total MET. (**E–G**) The data in (**B** and **F**) were quantified by densitometry analysis using Image J Software. Data represent the mean ± SEM of triplicate wells. Statistically significant differences are indicated (*p < 0.05, **p < 0.01).

To verify the direct effect of FYN expression on NE markers, our next set of experiments were performed using DU145 Fyn-EV (characterized by undetectable levels of FYN expression as shown in Supplementary Figure 1 and Figure 7A), and DU145 Fyn-SIL, that overexpress FYN (Figure 7A and 7D). Confirming our hypothesis, the expression of CHGA and CHGB were directly associated with the expression of FYN on DU145 cells, and it was independent of HGF stimulation (Figure 7B–7E). These findings strongly suggest that FYN regulates the expression of NE markers in prostate cancer cells. Also, the expression of FYN on these cells led to phosphorylation of MET, independent of HGF stimulation (Figure 7F and 7G). At this point, it is still unclear if the NEPC differentiation requires MET/FYN axis signaling, however these studies identify the direct association of FYN and NEPC phenotype.

## DISCUSSION

NEPC is an aggressive subtype of PCa that remains an urgent and growing clinical problem. It is universally recognized as a form of PCa that has a rapidly evolving natural history punctuated by aggressive clinical features including the appearance of visceral metastasis. In fact, most patients who develop NEPC survive less than 1 year after diagnosis [27, 28]. The biology of NEPC is a new and growing ara of interest. The biological drivers for growth, differentiation, and metastasis have not yet been clearly delineated, though several studies have raised both traditional and non-traditional biomarkers, and targets based on the better-studied and more common gastrointestinal forms of neuroendocrine tumors (NETs). These tumors are often identified by the presence of biochemical features indicating neuroendocrine differentiation such as CHGA, CHGB, SYP, and CD56. Non-gastrointestinal forms of neuroendocrine cancer, while bearing these features, do not necessarily have the same biology and hence may not have the same clinical behavior. Understanding the biological drivers of non-gastrointestinal NETs such as NEPC remains an important translational task. Our group has already demonstrated the capacity for FYN to drive metastasis and growth in prostate cancer [9]. The initial and current studies on FYN have focused on its impact on the biology of the PC3 line, which has been characterized as NEPC/SCPC [15]. A better characterization of mechanisms/factors implicated in NE differentiation is likely to lead to the identification of new targets. In the present study, we report the following: (1) FYN is overexpressed in NEPC; (2) knockdown of FYN has reduced the expression of NE markers and metastatic potential of NEPC, and; (3) FYN regulates expression of NE markers in three different PC cell lines.

To date, the standard of care for men with NEPCs remains the use of platinum based chemotherapy. This form of treatment is associated with commonly feared adverse events including nausea, vomiting, alopecia, neuropathy, and cytopenias given its diffuse and potent cytoxic effects. Despite its common use, however, there is little data to support its continued use in this subset of prostate cancers [3, 29]. As such, there continues to be an urgent need for improved therapeutics. At this time, a select few kinase inhibitors have been explored, but to date none have demonstrated clear activity or benefit (such as alisertib).

The Src-family kinases (SFKs) have been recognized as promising targets for cancer therapy given their capacity to synergize with other signaling pathways [9, 10, 30–33]. They have been particularly appealing targets given the availability of well-tolerated agents that can be used to suppress their activation such as dasatinib, saracatinib, and bosutinib. Treatment with SFK inhibitors has been associated with a number of biological effects including reduction of metastatic potential as exhibited *in vivo* [34]. Early pre-clinical and clinical studies with SFK inhibitors such as dasatinib showed potent biological effects [35, 36]. The subsequent Phase 3 study combining SFK inhibition (using dasatinib) with docetaxel chemotherapy in an unselected pool of men with mCRPC did not yield an improvement in overall survival [31]. This disappointing clinical result is presumably the consequence of two major caveats in the trial design. First, the clinical pairing with docetaxel may not have been optimal since no agent to date has shown the capacity to improve upon the taxane effect [37] Second, there is tremendous molecular heterogeneity in this patient group. It is likely that were patients with varying degrees of dependence on FYN or other SFKs, thus focusing on a more optimized clinical patient subgroup may lead to a clearer benefit (e.g. providing trastuzumab in a HER-2 negative breast cancer population versus as HER-2 overexpressing population).

Our findings suggest the possibility of a role for FYN inhibition in NEPC growth and visceral metastasis. RNA-sequencing data from NEPC patient tissues and treatment resistant NEPC PDX models reveal specific up-regulation of FYN kinase but not c-SRC or LYN [38]. These observations in human clinical samples reveal that FYN could be a potential biomarker and therapeutic target for NEPC. Refining patient selection based on the expression of FYN or related molecular signals (i.e. MET) especially in patients subgroups with NEPC features represents a more refined clinical approach that may have reveal the benefit of FYN inhibition. In addition, combining FYN inhibition with other therapeutics such as those which inhibit MET activation (e.g. cabozantinib) might also hold promise in the treatment advanced PCa and overcome limitation of single agent approaches. According to our results, FYN regulates the expression of MYCN suggesting that a combinatory therapy strategies targeting MYCN, FYN, and/or MET activation. Clearly, future experiments are required to elucidate the direct molecular mechanisms that drive NEPC activation and metastasis.

In summary, our studies demonstrate that FYN plays an important role in NEPC metastasis and progression in a xenograft tumor model. Our findings of FYN expression and function in NEPC are timely given and assume great significance due to the reported frequency of 25% of NEPC in advanced PCa patients who have *de novo* or emerging resistance to next-generation AR-targeted therapies. Thus, our findings demonstrate that FYN is an important biomarker and therapeutic target worthy of further exploration that may reshape care for men in NEPC.

## MATERIALS AND METHODS

### Cell lines and reagents

PC3 cells were a generous gift from Dr. Carrie Rinker-Schaeffer. TRAMPC2, DU145, and LNCaP cells were purchased from ATCC, and ARCaP_M_ obtained from Novicure Biotechnology. PC3 and ARCaP_M_ cell line variants were generated as previously described [9]. Antibodies used for Western blot analysis or multiplexed quantum dot labeling (mQDL) were: anti-FYN antibody (Cell Signaling #4023 or Santa Cruz #sc16), phospho-MET (#3126) and total MET (#4560) obtained from Cell Signaling. Anti-CD44 (sc-7297); anti-CD56 (sc-7326); anti-CHGA (sc-13090), anti-SYP (sc-17750), all obtained from Santa Cruz. Other common reagents for mQDL were used as described previously [39, 40]. Recombinant human HGF was purchased from Calbiochem (Millipore).

### Cell culture

TRAMPC2 and ARCaP_M_ cells were cultured in T-medium (GibcoBRL) supplemented with 5% heat inactivated fetal bovine serum (FBS; Omega Scientific, Inc). PC3, LNCaP and DU145 were cultured in RMPI 1640 with 10% FBS. Each had 50 IU/mL penicillin and 50 μg/mL streptomycin (GibcoBRL) in 5% CO_2_ at 37°C. All cells were negative for mycoplasma contamination (MycoAlert Mycoplasma Detection kit from Lonza).

### Lentiviral transduction

*FYN*-altered lines were generated as previously described by our group [9]. In brief, PC3 cell lines were transduced with lentivirus with an shRNA targeted against *FYN* (FYN-) or a GC-content matched, non-targeting shRNA control (NT), each construction containing puromycin resistance gene. Lentiviral preparation and transduction of cell lines were performed as per the manufacturer's instructions (Sigma Aldrich, St. Louis, MO). Cells were selected in puromycin (for FYN shRNA) before experiments were performed. A rescue/overexpressing PC3 and DU145 lines were created using another lentivirus containing a *FYN* construct with a silent mutation to avoid the shRNA effect from knockdown named FYN-SIL. The corresponding empty vector control for the FYN- background was called FYN-EV. Both constructs contained a blasticidin resistance gene. TRAMPC2 cells were transduced with a retroviral vector with RANKL gene cloned into the pIRES-GFP-Puromycin vector and cells were selected for puromycin expression.

### Histopathology and Multiplexed QD Labeling (mQDL)

We performed mQDL procedures, multi-spectral image acquisition, signal unmixing and quantification as described in our published protocol [13]. The cell or tissue specimens were subjected to sequential labeling of CD44, FYN, CD56 and CHGA or SYP as follows: 1) ant-CD44 (1:100), QD655 (1:100), 2 hr at 37°; 2) anti-FYN (1:50), QD605 (1:100), overnight at 4°; 3) ant-CD56 (1:50), QD625 (1:150), 2 hr at 37°; and 4) anti-CHGA or SYP (1:100), QD585 (1:100) overnight at 4° and completed with 4′6-diamidino-2-phenylindole (DAPI) mounting (Vector Laboratories) for imaging. Negative controls were performed in parallel by replacing the species and dilution matched immunoglobulin subtypes applied to an immediately adjacent tissue section. Image acquisition and deconvolution or unmixing, signal quantification, and statistical analyses were performed as described previously [12, 13, 15, 16]. Human tissues analyzed in this study were collected and characterized under CSMC IRB-approved protocols after obtaining informed consent in compliance with the Declaration of Helsinki. Usage of clinical specimens was approved by the Institutional Research Board (IRB# Pro00025216).

### Correlation analysis of FYN and NE markers in human PCa

To compute Spearman's correlation coefficients between FYN and NE markers such as Neuron specific enolase (NSE), Chromogranin A (CHGA), Chromogranin B (CHGB), Aurora Kinase A (AURKA), N-Myc (MYCN), and Secretogranin 3 (SCG3). We used global gene expression profiles derived from human PCa tissues. Four independent datasets [18–21] were downloaded from Gene Expression Omnibus (GEO) database. These datasets were selected by the criteria that each dataset contains more than 50 samples of primary PCa. The intensities in each dataset were normalized by quantile normalization method [41]. Given the normalized intensities of the whole genes in the dataset, the intensities of FYN, NSE, CHGA, CHGB, AURKA, and SCG3 in primary PCa samples were extracted and utilized to compute Spearman's correlation coefficients.

### Growth, invasion and migration assays

ARCaP_M_ were grown in 12 well plates and counted on day 4. PC3 NT and FYN invasion assay was performed in the BD BioCoat tumor invasion system (24 multi-well plate with 8 μm; BD Biosciences) according to the manufacture's instructions. ARCaP_M_ NT and FYN were starved on T medium 0.1% BSA. 1 × 10^5^ cells were applied to chamber well 8-micron. The bottom chamber contained medium with or without 50 ng/ml of rhHGF. Cells were incubated for 48 h and the cells that had invaded and attached to the lower surface of the membrane was fixed and stained with hematoxilin.

### Quantitative PCR

Total RNA was isolated from confluent monolayers of cells using the RNeasy Mini Kit (Qiagen) RNA was converted to cDNA using Superscript^®^III reverse transcriptase (Life Technologies) or iScript^™^ cDNA Synthesis Kit (Bio-Rad). Messenger RNA expression levels were determined RT-PCR assay and SYBR Green Dye (Applied Biosystems), and mRNA expression was normalized to *GAPDH*. The fold change in transcript expression was calculated over the expression of non- targeting shRNA control (NT) or empty vector (FYN- EV) without stimulation. *CHGA, CHGB, SYP, AURKA, MYCN* primers were designed and synthesized at Integrated DNA Technologies. *FYN* primer was purchased from Applied Biosystems.

### *In vivo* metastasis assay

The bioluminescent human PC3 and mouse TRAMPC2 prostate carcinoma cell lines were generated by stable retroviral transduction of MSCV-Luc-Hygro vector. PC3 NT or PC3 FYN- cells (0.5 × 10^6^) were injected via intracardiac route into SCID mice (Strain Code: 236, Charles River Laboratories). The TRAMP2 control and TRAMPC2-RANKL cells were injected via intracardiac route into C57BL/6 mice (Jackson laboratories). Briefly, cells were injected into the left ventricle of the heart as an experimental metastasis model. Mice were monitored on a weekly basis by bioluminescence imaging for *in vivo* growth of tumors and metastasis after injection for 4 weeks. For histological analysis, excised lung samples were fixed with 10% buffered formalin and processed using routine histological techniques. Tissue sections were stained with H&E. The Cedars-Sinai Medical Center Institutional Animal Care and Utilization Committee approved all protocols regarding animal procedures and care.

### Statistics

Statistical differences were detected using the unpaired Student's *t*-test. Values are presented as mean ± SEM. *P* values ≤ 0.05 were considered statistically significant. For *in vivo* metastasis studies, Kaplan-Meier Survival Curve Analysis was applied to assess the differences in the survival between the two groups of mice. Calculations were performed using the Prism 5.0 software program for Apple Computers (GraphPad Software).

## SUPPLEMENTARY MATERIAL FIGURES


