# Supplementary material for: SRC family kinase FYN promotes the neuroendocrine phenotype and visceral metastasis in advanced prostate cancer
Source: Oncotarget. 2015 Nov 26;6(42):44072–83. doi: 10.18632/oncotarget.6398 (PMC4792542; doi:10.18632/oncotarget.6398)
Supplement: Supplementary file 1 [file oncotarget-06-44072-s001.pdf]

SRC family kinase FYN promotes the neuroendocrine phenotype and visceral metastasis in advanced prostate cancer

Supplementary Materials

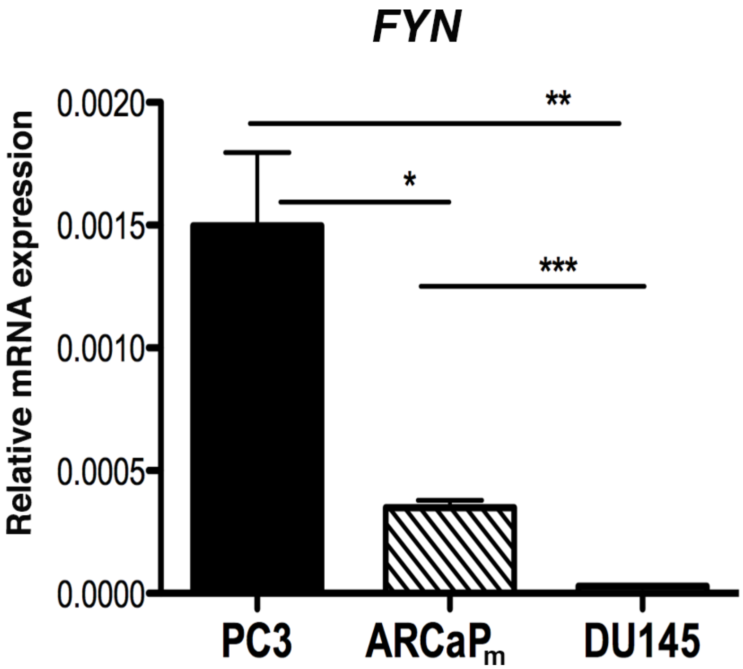

Supplementary Figure S1: Relative mRNA *FYN* expression in PC3, ARCaP$_M$, and DU145 PCa cell lines. *FYN* expression was determined by RT-PCR. Data are representative of two independent experiments performed in individual triplicates (mean ± SEM). Statistical differences are indicated (*$p < 0.05$, **$p < 0.01$, ***$p < 0.0001$).

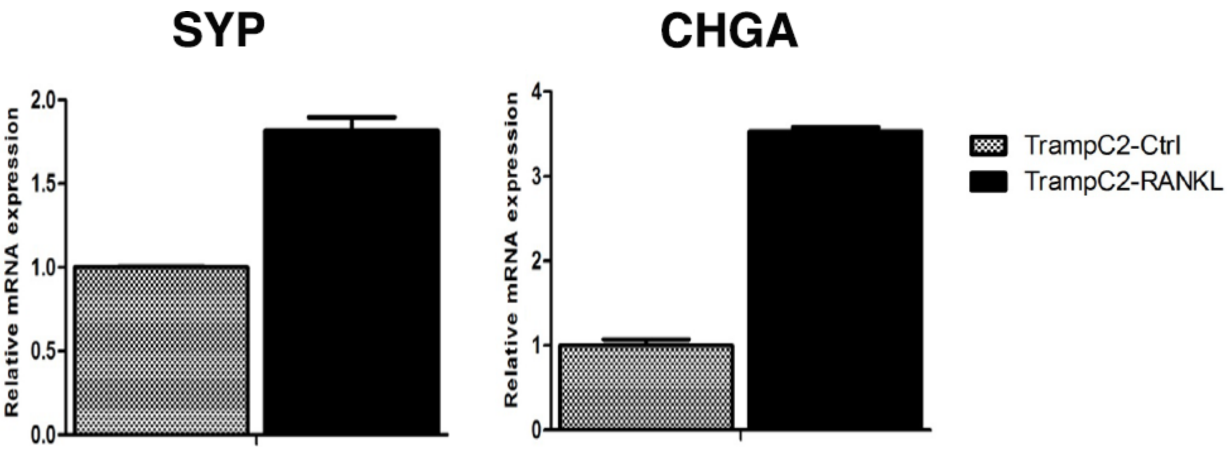

Supplementary Figure S2: High levels of NE markers on TRAMPC2-RANKL cells. Transcript levels of *CHGA* and *SYP* by Taqman.
